# Wearable Sensors and Machine Learning for Hypovolemia Problems in Occupational, Military and Sports Medicine: Physiological Basis, Hardware and Algorithms

**DOI:** 10.3390/s22020442

**Published:** 2022-01-07

**Authors:** Jacob P. Kimball, Omer T. Inan, Victor A. Convertino, Sylvain Cardin, Michael N. Sawka

**Affiliations:** 1 School of Electrical and Computer Engineering, Georgia Institute of Technology, Atlanta, GA 30332, USA; inan@gatech.edu; 2 Battlefield Health & Trauma Center for Human Integrative Physiology, US Army Institute of Surgical Research, JBSA Fort Sam Houston, San Antonio, TX 78234, USA; victor.a.convertino.civ@mail.mil; 3 Naval Medical Research Unit, JBSA Fort Sam Houston, San Antonio, TX 78234, USA; sylvain.cardin.civ@mail.mil; 4 School of Biological Sciences, Georgia Institute of Technology, Atlanta, GA 30332, USA; michael.sawka@biosci.gatech.edu

**Keywords:** wearable sensors, compensatory reserve, cardiac decompensation, dehydration, physical work capabilities, environmental stress and adaptation

## Abstract

Hypovolemia is a physiological state of reduced blood volume that can exist as either (1) absolute hypovolemia because of a lower circulating blood (plasma) volume for a given vascular space (dehydration, hemorrhage) or (2) relative hypovolemia resulting from an expanded vascular space (vasodilation) for a given circulating blood volume (e.g., heat stress, hypoxia, sepsis). This paper examines the physiology of hypovolemia and its association with health and performance problems common to occupational, military and sports medicine. We discuss the maturation of individual-specific compensatory reserve or decompensation measures for future wearable sensor systems to effectively manage these hypovolemia problems. The paper then presents areas of future work to allow such technologies to translate from lab settings to use as decision aids for managing hypovolemia. We envision a future that incorporates elements of the compensatory reserve measure with advances in sensing technology and multiple modalities of cardiovascular sensing, additional contextual measures, and advanced noise reduction algorithms into a fully wearable system, creating a robust and physiologically sound approach to manage physical work, fatigue, safety and health issues associated with hypovolemia for workers, warfighters and athletes in austere conditions.

## 1. Introduction

Hypovolemia is a physiological state of reduced blood volume that impairs physical work capability [1], cognitive-motor function [2], and environmental stress tolerance [3,4]. Hypovolemia also increases risks for orthostatic intolerance [5], occupational accidents [6], circulatory collapse and shock [7] and contributes to a variety of health issues [8]. Accordingly, hypovolemia is ubiquitous in occupational, military and athletics applications, and can impact workers, warfighters and athletes alike. Historically, each hypovolemia problem has been viewed independently, and mitigation approaches included providing general guidance regarding fluid replacement, managing environmental exposure, employing work–rest periods or attempting wearable physiological monitoring. Previous wearable physiological monitoring approaches were based on easily measured conventional vital signs (e.g., body temperature, heart rate) often related to the specific exposure in the field [9,10,11]. However, measures of standard vital signs are limited in their ability to provide individual-specific information about those at greatest health risk or with performance impairments because they are not based on the understanding of underlying integrated physiological mechanisms associated with the adverse outcomes from hypovolemia. Compensatory reserve and decompensation measures demonstrate great clinical promise for monitoring and treating hemorrhage hypovolemia [7,12], and we argue these approaches can be effectively applied to a broader set of hypovolemia problems [13]. However, the broader use of compensatory reserve or decompensation measures, particularly in austere field conditions where multiple stressors are combined with hypovolemia, pose several technology and algorithmic challenges that preclude current approaches from translating successfully to field settings.

We previously reported the significance of measuring the compensatory reserve as a tool for advanced decision support in the clinical setting of life-threatening hemorrhage [14]. In this paper, we extend our previously published review by examining the physiology of hypovolemia and its association with health and performance problems common to occupational, military and sports medicine. We will discuss the maturation of compensatory reserve and decompensation measures so future wearable sensor systems can be utilized to effectively manage these hypovolemia problems. This manuscript is a companion to and expands on our previously published review regarding applying compensatory reserve and decompensation measures to the clinical problem of hemorrhage [14].

## 2. Hypovolemia and Cardiovascular Adjustments

### 2.1. Relative and Absolute Hypovolemia

**Figure 1:**
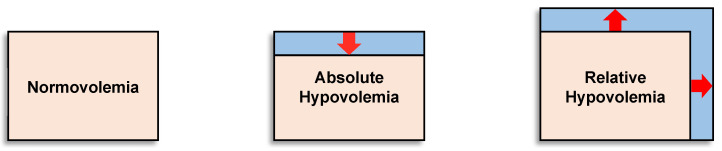
Illustration of the concept of absolute and relative hypovolemia. Pink represents the vascular (blood) volume and blue represents the vascular space. Absolute hypovolemia (reduction in blood/plasma volume) can be mediated by factors such as hemorrhage or dehydration; relative hypovolemia can be mediated by factors that increase vascular space such as increased cutaneous vasodilation from heat stress, hypoxia, intense physical exercise, or systemic vasodilation from sepsis. Image modified from [15].

Hypovolemia may exist as either absolute hypovolemia because of a lower circulating blood (plasma) volume for a given vascular space (e.g., dehydration, hemorrhage) or relative hypovolemia resulting from an expanded vascular space (e.g., vasodilation of skin or skeletal muscle) for a given circulating blood volume (e.g., heat stress, hypoxia, sepsis). It is important to recognize that hypovolemia represents a compromise to an individual’s capacity to compensate for conditions of low circulating blood volume or flow. In many situations, both absolute and relative hypovolemia occur simultaneously, thus synergistically augmenting the circulatory and metabolic problems for a given level of blood volume reduction or systemic vasodilation (expanded vascular space), which can adversely impact health and performance. Figure 1 illustrates the concept of normal blood volume (normovolemia), absolute hypovolemia and relative hypovolemia. Factors mediating hypovolemia, such as decreased blood or plasma volume, increased vascular space and decreased total body water (dehydration), can change dynamically with multiple perturbations (e.g., hemorrhage, sickness, hydration status, heat stress, cold and hypoxia) and impair health and performance. Conversely, it will be presented that important physiological adaptations from physical training and heat acclimation include blood volume expansion, increased stores of oxygen in the body, and vascular changes that minimize hypovolemia and contribute to performance improvements.

### 2.2. Hypovolemia from Dehydration

**Figure 2:**
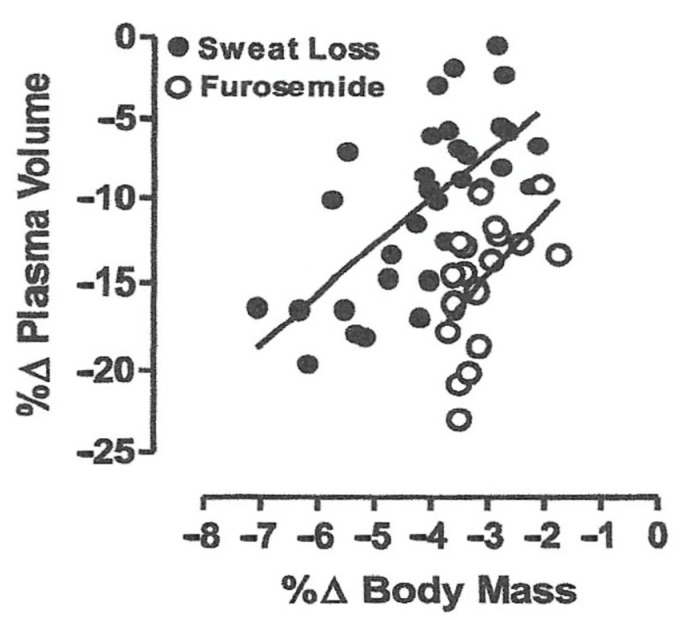
Linear regression of plasma volume loss (hypovolemia) and body water deficit (percent change in body mass) relationship for hypertonic (sweat loss) and isotonic (Furosimide diuretic) dehydration [16].

A body water deficit >2% of body mass (or ~3% of total body water) is defined as dehydration and can occur from sweat loss in warm or hot conditions and/or diuresis from cold, hypoxia and aquatic environment exposure or from sickness that causes vomiting or diarrhea [8,16]. Dehydration elicits intracellular and extracellular water loss proportional to water and solute deficits [8,16]. An iso-osmotic mediated hypovolemia (from cold, high-altitude and aquatic exposure) results in greater plasma loss (and thus blood volume reduction) for a given water deficit than hypertonic mediated hypovolemia from sweat loss [16]. Plasma (the liquid portion of blood) accounts for ~50 to 60% of blood volume, with the remainder represented by circulating cells. The difference in plasma loss between isotonic and hypertonic dehydration can be explained by two factors: (1) an elevation in intravascular osmotic pressure with hypertonic hypovolemia (less solute loss) pulls intracellular water from tissue into the vascular space; and (2) substantial extracellular solute (e.g., sodium, chloride) losses with isotonic hypovolemia translate to proportionate fluid loss from both plasma and total body water [16]. Figure 2 demonstrates the impact of a given body water deficit (based on body mass loss) on the magnitude of hypovolemia (presented as percent change in plasma volume) from hypertonic (sweat loss) and isotonic (Furosemide) mediated dehydration [16]. Note the isotonic dehydration (Furosemide) elicited a greater plasma loss for a given body water deficit (dehydration level). Thus, the dehydration-mediated hypovolemia and decreased preload are dependent upon the magnitude and type of dehydration. Dehydration-mediated hypovolemia can often be mixed by exposure to combinations of physical work, environmental stress and sickness perturbations.

### 2.3. Hypovolemia from Environmental Stress

Hypovolemia is common with exposure to heat [1], cold [17], high-altitude [18] and aquatic [19] environments, and can impair subsequent physical work capabilities and accentuate several health issues. During physical work in the heat, the most significant physiological burden is the cardiovascular support of high skin blood flow for heat dissipation while recruitment of compensatory mechanisms attempts to maintain adequate blood pressure to perfusion tissues [20]. Warm–hot skin is associated with a greater cutaneous vasodilation (skin blood flow) and venous compliance (skin blood volume), which displaces blood away from the central circulation augmenting cardiovascular strain [1,20,21]. This increase in vascular space is often associated with a concurrent sweat loss-mediated hypertonic dehydration, which decreases blood (plasma) volume [20]. In addition, high skin blood flow during heat stress is associated with plasma protein loss, which further augments the hypovolemia [22]. The dual perturbation of a reduced blood volume (absolute hypovolemia) with increased skin blood flow (relative hypovolemia) can reduce the ability to sustain cardiac output [23] and is an important physiological prerequisite to impair exercise capabilities and induce heat exhaustion [1,21,24]. The warmer the environment, the greater the impaired physical work capabilities for a given dehydration level [1].

Conversely, blood (plasma) volume expansion is an important heat acclimation adaptation that helps minimize cardiovascular strain and enable better sustainment of physical work capabilities [25]. Plasma volume expansion of 10% to 20% is commonly associated with short-term (1–2 weeks) acclimation to physical exercise, heat or a combination of both [25,26,27,28]. Thus, these physiological adaptations to heat acclimation should moderate the effects of subsequent hypovolemia. A recent study has demonstrated that long-term (5.5 weeks) exercise-heat acclimation increased blood volume by expanding both plasma volume and erythrocyte or the red blood cell portion of volume [29]. Depending upon whether the blood volume expansion was induced by plasma volume or erythrocyte volume expansion, the impact on abating the impact of subsequent hypovolemia will likely differ, with the latter being more effective [30].

During high-altitude exposure, there is a marked blood (plasma) volume reduction (absolute hypovolemia) that is proportionate to the elevation ascended and duration of residence [18]. This plasma volume reduction is primarily due to diuresis (isotonic dehydration) as well as the loss of total circulating plasma protein [31,32,33]. For example, at 2500 m (~8200 feet) plasma volume can decrease by ~10% on day 3 and by ~13% on day 6 of residence [18]. In addition, physical work at high-altitude induces sweat rates comparable to those at sea-level for a given exercise-heat strain [34], while respiratory water loss is elevated [31], both contributing to dehydration. Furthermore, acute high-altitude exposure can induce cutaneous vasodilation or relative hypovolemia [35]. Hypovolemia contributes equally to hypoxia in impairing physical work capabilities at high-altitude and may contribute towards symptomatology of Acute Mountain Sickness [3].

During cold stress there is a marked diuresis (isotonic dehydration) and blood (plasma) volume reduction which is not altered by acclimatization status [17,36] For example, whole-body cooling decreases plasma volume by ~12% in air and ~17% with water immersion [17]. It is important to note that simultaneous cold and water immersion accentuates the magnitude of hypovolemia, and these stressors often appear in concert. Cold exposure causes peripheral vasoconstriction [37], reducing the vascular capacity, thus eliciting diuresis and isotonic dehydration through the loading of central baroreceptors [26]. In cold environments, hypovolemia issues typically occur if subsequently performing physical work while wearing warm clothes or protective equipment thus inducing heat strain [36], which induces a relative hypovolemia (skin and skeletal muscle vasodilation) upon the previously suffered absolute hypovolemia. In aquatic environments (e.g., swimming and diving), exposure to hydrostatic effects induces marked diuresis and isotonic dehydration through the loading of central baroreceptors [19]. Hypovolemia problems typically occur when transferring from an aquatic to land environment and attempting physical work, which induces a relative hypovolemia upon the previously suffered absolute hypovolemia.

### 2.4. Hypovolemia from Physical Work

Physical work increases the metabolic demands within active skeletal muscle, which induces vasodilation and reduction in total peripheral resistance. The drop in total peripheral resistance is proportional to the metabolic rate. To meet the metabolic demands of active skeletal muscles, cardiac output increases with work intensity as a product of elevated heart rate and stroke volume [38]. Thus, physical work induces an acute relative hypovolemia due to active skeletal muscle dilation and, if heat strain is present, a concurrent cutaneous vasodilation. With physical training, blood volume expansion translates to greater filling of the cardiac ventricles, increased maximal cardiac output and improved physical work capabilities [38,39]. An analysis of composite data from 18 physical training studies demonstrated ~10% increase in blood volume over the initial several weeks [40]; but some studies have reported much larger expansions, such as ~25% [38]. It is important to note that the blood volume expansion is initially due to a plasma volume expansion over the initial few weeks, but subsequently that expansion retracts as erythrocyte volume expands during the following weeks [40]. Likewise, in addition to blood volume expansion there is an increased vascular space due to increased capillarization and arterial remodeling with physical training [38]. To achieve greater cardiac output with improved physical work capabilities, there are cardiac hypertrophy and cardiac function improvements. All of these cardiovascular adaptations from physical training follow different time courses but are dependent upon each other to achieve greater cardiac output and contribute to the improved physical work capacity [38]. Likewise, with physical inactivity and detraining, the blood volume contracts and is partially responsible for a fall in maximal cardiac output and physical work capabilities [39,41].

### 2.5. Cardiovascular Adjustments to Hypovolemia

**Figure 3:**
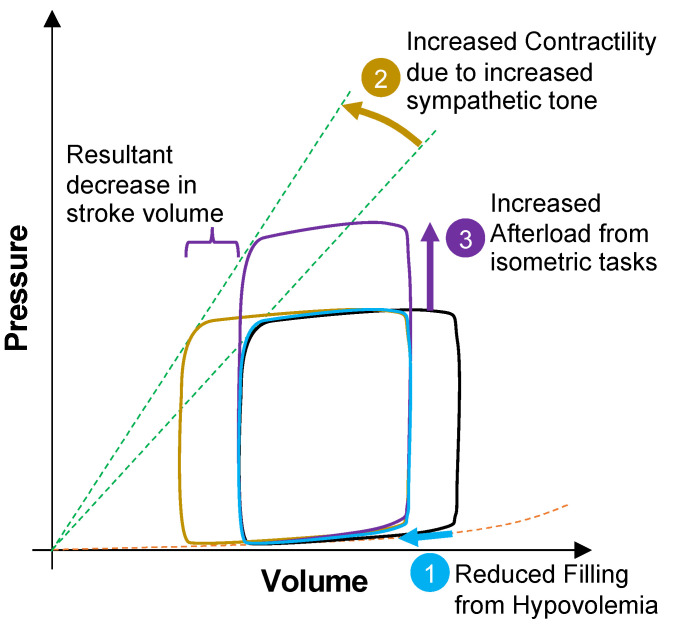
Ventricular function curves showing the sequence of cardiovascular events during hypovolemia while performing an occupational work activity. Hypovolemia causes reduced ventricular filling or preload (1). Then, the body compensates by increased sympathetic tone, resulting in elevated heart rate and cardiac contractility (2). During physical activity, the worker periodically performs upper limb isometric tasks, thus increasing afterload (3). The result is decreased stroke volume and increased myocardial oxygen demands.

Cardiovascular adjustments imposed by absolute and/or relative hypovolemia have many commonalities, such as reduced cardiac filling, altered cardiac mechanics, arterial pressures and arterial pressure waveforms. Figure 3 shows ventricular function curves describing the cardiovascular problems imposed by hypovolemia while performing an occupational work activity and then with a simultaneous isometric task. With hypovolemia, there is reduced cardiac right atrial pressure, reduced cardiac ventricle filling (preload), increased contractility and falling stroke volume with an elevating heart rate [7,20]. As hypovolemia becomes more severe, cardiac output and blood pressure regulation are challenged because reduced diastolic filling lowers end-diastolic volume and reduces stroke volume, and consequently an elevated heart rate and cardiac contractility are required to maintain cardiac output. However, an elevated heart rate implies that the cardiac cycle is shortened, and this will lower the time for diastolic filling, which may further compromise stroke volume and cardiac output [42]. A reduced cardiac output results in difficulty to sustain the arterial blood pressure required for adequate tissue perfusion needed to support performance. If the worker subsequently grasps a tool, an isometric reflex then occurs which increases blood pressure, afterload and heart rate [43,44]. The reduced cardiac filling from hypovolemia combined with increased afterload (from isometric and upper body exercise) imposes a burden on the myocardium from increased oxygen demands while working on an inefficient portion of the ventricular function curve, a condition that will further reduce ejection fraction and increase the decline in cardiac output. Together, these factors make it difficult to sustain the required cardiac output for tissue and organ perfusion, and for workers with advanced heart disease, may potentially induce angina or myocardial infarction.

**Figure 4:**
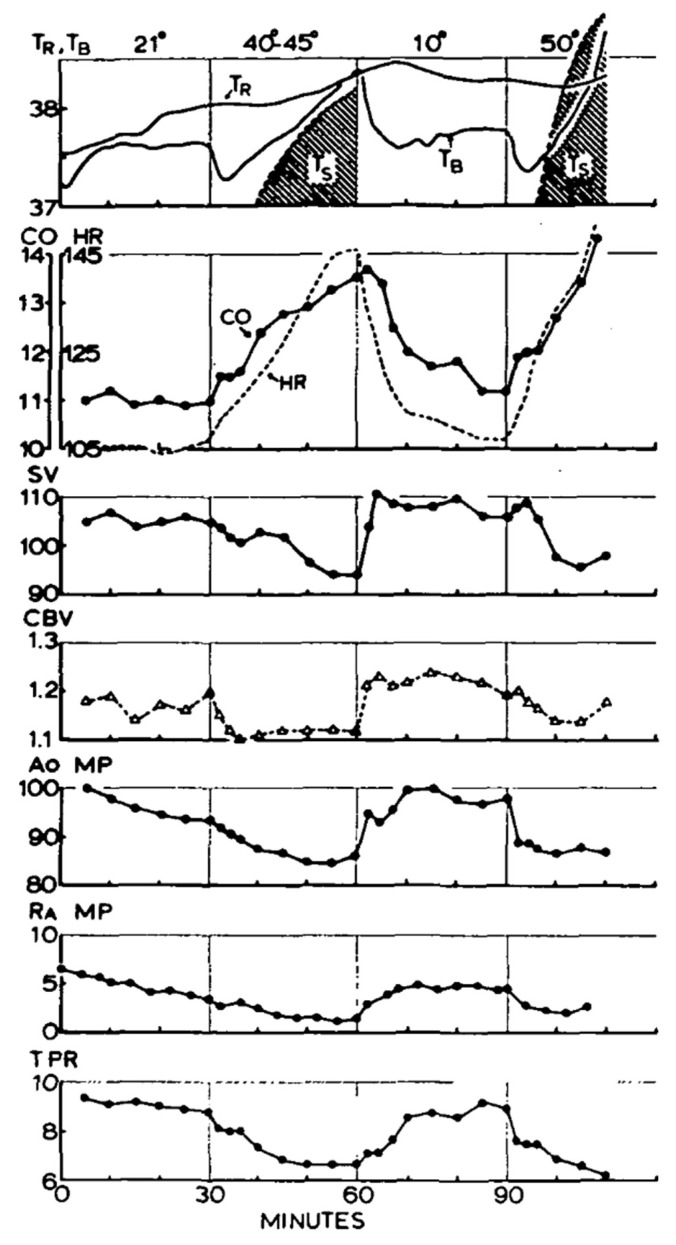
Demonstration of the impact of skin warming on skin temperature (T_s_), rectal temperature (T_R_),blood temperature from right atrium (T_B_), total peripheral resistance (TPR), right atrial mean pressure (R_A_MP), aortic mean pressure (AoMP), central blood volume (CBV), stroke volume (SV), and heart rate (HR) and cardiac output (CO). Image from [45].

Figure 4 provides cardiovascular data demonstrating the impact of relative hypovolemia mediated by experimentally elevating skin temperature that resulted in cutaneous vasodilation [45]. With skin (T_s_)warming, the total peripheral resistance (TPR), right atrial mean pressure (R_A_MP), aortic mean pressure (A_o_MP), central blood volume (CBV) and stroke volume (SV) decrease, while heart rate (HR) is elevated to sustain cardiac output (CO). During heat exposure, this relative hypovolemia often can be coincident with dehydration (absolute hypovolemia). The combination of both relative and absolute hypovolemia in conditions of hyperthermia will accentuate the reduced cardiac filling and subsequently reduce cardiac output. As the metabolic demand for physical work increases, a progressive cardiac output reduction (relative to control conditions) occurs due to greater vasodilation in working skeletal muscles [23]. The hypovolemia-mediated reduction in cardiac output translates to an inability to sustain blood pressure [21] as the left ventricular function is sustained [46]. Although cardiac afterload does not increase with relative hypovolemia due to peripheral vasodilation, a marked increase in blood pressure is likely to occur if an isometric task like gripping a tool or weapon is simultaneously performed. With absolute hypovolemia, a compensatory elevation in sympathetic nerve output results in peripheral vasoconstriction. The resulting elevation in peripheral vascular resistance can increase arterial blood pressure (afterload), but this is more likely with hyperosmotic hypovolemia dehydration because of a marked influence of elevated osmolality on sympathetic nervous activity [47]. However, if dehydration (absolute hypovolemia) occurs with marked heat stress (relative hypovolemia), the cutaneous vasodilation will offset the increased sympathetic output for compensatory vasoregulation (decrease gut blood flow) and not alter or decrease afterload.

## 3. Identifying Integrated Physiological Signals of Compensatory Reserve or Decompensation

For many occupational, military and athletic situations, an individual may suffer marked hypovolemia, which impairs health, safety and performance. As discussed, hypovolemia can occur from dehydration or decreased total circulating protein and/or increased vascular space from cutaneous and skeletal muscle vasodilation induced by environmental exposure or performing physical work. It is important to note that the physiological responses to these conditions are highly individual, with some people exhibiting much greater tolerance and capacity to compensate for the conditions than others [4,48,49].

Decision-support wearable technologies are needed that can measure the integrated physiological compensation or decompensation providing ‘individualized’ assessment of progression towards hypovolemia-mediated compromised capacity, or degree of physiological adaptation to several stressors that protect against hypovolemia to sustain performance [7,13,14]. A ruggedized wearable physiological monitoring system that can reliably measure the magnitude of integrated physiological compensation or decompensation from hypovolemia would provide critical information to manage health, safety and optimize performance [13].

### 3.1. Compensatory Reserve

**Figure 5:**
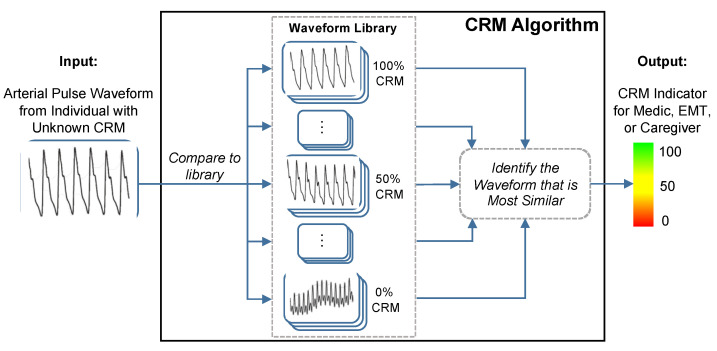
The conceptual framework of the compensatory reserve measure (CRM) algorithm. The input waveform from the current subject is compared to a library of more than 650,000 waveforms recordings collected from more than 260 subjects exposed to experimentally-controlled progressive reductions in central blood volume by lower-body negative pressure to generate an estimated individual compensatory reserve measurement (CRM). Image modified from [50].

The compensatory reserve measure (CRM), a novel concept introduced by Convertino and colleagues, provides a single indicator, measured peripherally with noninvasive sensors, that could represent the sum of compensatory responses to hypovolemia and a validated index of potential cardiovascular instability [7]. The CRM uses a deep convolutional neural network to compute the distance or similarity between recorded vascular signal segments from either a non-invasive continuous blood pressure waveform or a transmissive photoplethysmogram (TPPG) waveform to a library of arterial waveforms recorded from subjects with known CRM as shown in Figure 5 [50]. The label from the library waveform with the closest distance or highest similarity to the incoming waveform is then assigned as the prediction value for the incoming waveform. The library used for comparisons contains noninvasive blood pressure waveforms recorded from more than 260 healthy subjects (men and women aged 18 to 55 years) who underwent graded lower body negative pressure (LBNP) to induce central hypovolemia until they reached a point of decompensated shock, which was labeled as 0% compensatory reserve [13]. “Decompensated shock” refers to the point at which the ongoing trauma or stress to the body overwhelms the body’s compensatory measures. For the CRM, 0% or “decompensated shock” was defined as the point during the LBNP protocol at which the subject experienced presyncope, indicating inadequate blood circulation to the brain. The subject’s normal baseline is then defined as 100% CRM, during which their body is not under any strain. The CRM’s performance in detecting and monitoring hypovolemia due to hemorrhage has been well documented [7] and we will present data demonstrating its sensitivity to heat stress, dehydration and physical exercise.

### 3.2. Validation of Compensatory Reserve for Heat Stress, Dehydration and Physical Exercise

**Figure 6:**
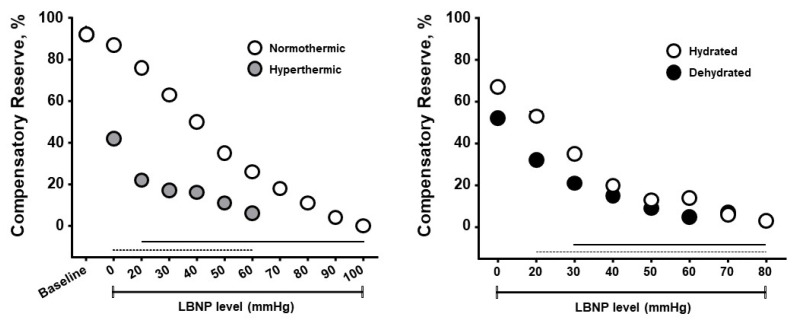
Compensatory reserve measures for normothermic vs hyperthermic subjects (**left**) and euhydrated vs dehydrated subjects (**right**) during progressive lower body negative pressure (LBNP) experiments. Data are means and 95% confidence intervals, with the solid lines at the bottom indicating statistically significant differences from baseline. Image modified from [13].

The compensatory reserve measure has been shown to be sensitive to hypovolemia induced by heat stress, physical exercise, dehydration, resting recovery and rehydration. Figure 6 presents results from experiments designed to determine if CRM differences could be observed with whole-body hyperthermia (heat stress) and if such differences would correspond to decreased tolerance to progressive hypovolemia induced by lower body negative pressure (LBNP) [51]. Healthy subjects underwent LBNP when normothermic (core temperature 37 °C) and hyperthermic (core temperature 38.3 °C), and after sweat-induced dehydration of 2% of their body mass. Mean baseline CRM were 92% on both days, however the cutaneous vasodilation during hyperthermia was associated with <50% in baseline CRM with a more rapid cardiovascular collapse. These data demonstrate that CRM is sensitive to relative hypovolemia induced by hyperthermia. During the euhydration (hydrated) and dehydration experiments all subjects were hyperthermic (core temperature 38.2 °C), thus experiencing relative hypovolemia (cutaneous vasodilation from hyperthermia) or relative with absolute hypovolemia (dehydration). CRM was initially lowered with dehydration compared to euhydration, and with LBNP the dehydrated subjects demonstrated a lower CRM with a more rapid onset of cardiovascular collapse (i.e., reduced physiological performance). Interestingly, the impact of dehydration with hyperthermia on CRM between experiments was initially more modest than with hyperthermia alone. These data demonstrate that CRM is sensitive to relative and absolute hypovolemia and their additive effects with increasing LBNP causing greater simulated hypovolemia.

**Figure 7:**
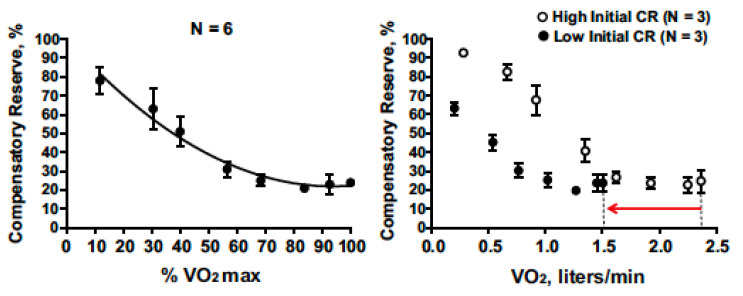
Compensatory reserve measure responses to progressive increases in aerobic exercise intensity (percent maximal aerobic power) that result in maximal exertion (**left**). On the (**right**), low baseline CRM (filled circles with 95% confidence intervals) is associated with lower maximal aerobic power (VO2max) compared to subjects with high initial CRM (open circles with 95% confidence intervals) with the final difference shown by the red arrow on the x-axis. Image from [13].

Several studies have demonstrated that CRM changes are sensitive to vasodilation and cardiovascular perturbations associated with physical exercise [52,53]. Figure 7 presents compensatory reserve values from subjects during progressive intensity cycle ergometer exercise until they achieved their maximal oxygen uptake (VO_2max_). CRM progressively decreased with increasing exercise intensity to an asymptote at ~20%. This response is logical as a greater oxygen uptake should translate to greater active vasodilation or relative hypovolemia. The asymptote at 20% suggests that blood pressure regulation was not the limiting factor for maximal intensity exercise.

It is reasonable to anticipate that if this exercise was performed during heat stress conditions, CRM would have deceased further indicating muscle oxygen delivery as a more limiting factor.

**Figure 8:**
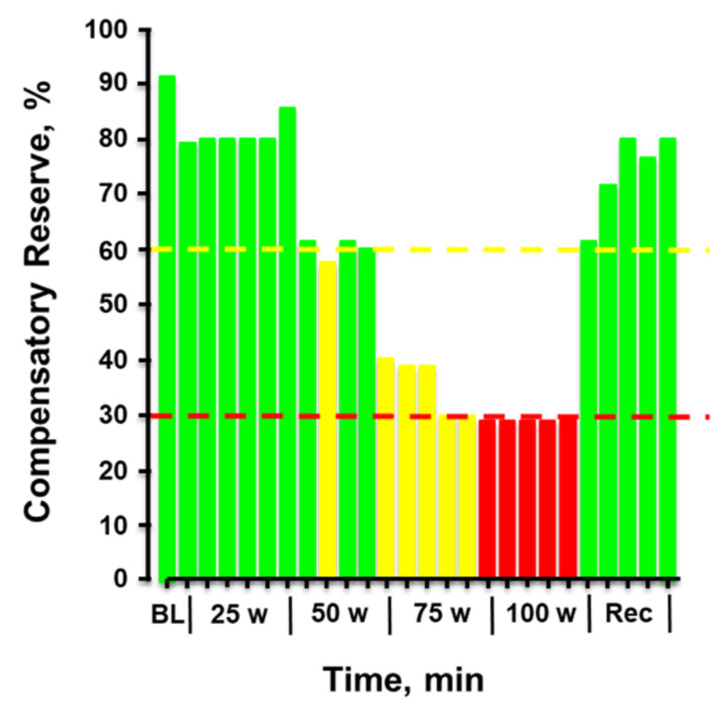
Compensatory reserve measured in a human subject during a 20-min graded cycle ergometer exercise performed at 100 °F air temperature. Each bar represents the average response over 1 min. Bar colors: green, compensatory reserve >60%; yellow, compensatory reserve ≤60% and >30%; red, compensatory reserve ≤30%. BL, baseline; W, watts. Image modified from [52].

Figure 8 provides the plotted measurements of compensatory reserve influenced by simultaneous exposure to physical exercise with heat stress and then resting recovery. In this figure, a human subject performed progressively increasing levels of physical exercise in a hot environment of 100 °F air temperature. Note the dramatic progressive reduction in compensatory reserve from a resting value of 91% in a room controlled at 75 °F air temperature to a significantly compromised level of <30% after only 20 min exposure to exercise and heat. After exercise was terminated and the subject recovered in the hot conditions, the compensatory reserve was restored to nearly 80%, suggesting that ~50% of the capacity to compensate for hypovolemia was attributed to the metabolic demand (active muscle vasodilation) of physical exercise while the remaining ~10% could be explained by the cutaneous vasodilation induced by heat. In this regard, a measurement of compensatory reserve provides an accurate integrated indicator of the individual’s physiological status for continued successful performance.

**Figure 9:**
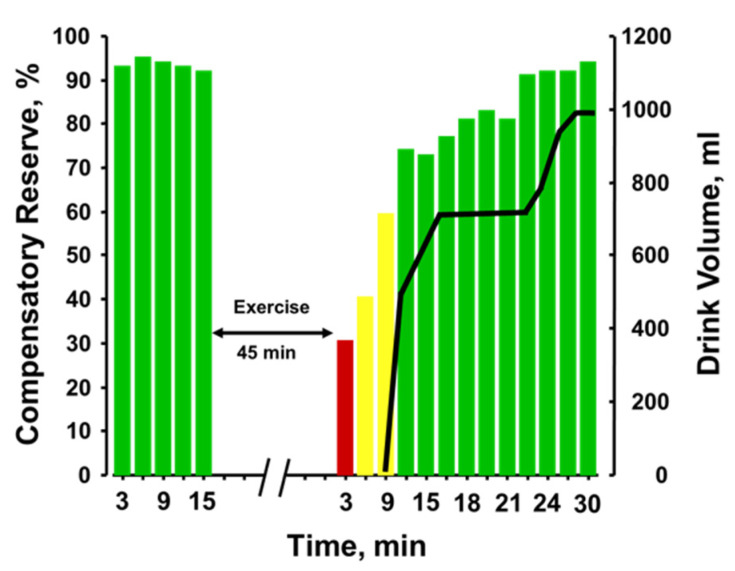
Compensatory reserve measures before and after 45 min of running exercise in the heat and resting recovery (10 min) and then fluid replacement (black line). Bar colors: green, compensatory reserve >60%; yellow, compensatory reserve ≤60% and >30%; red, compensatory reserve ≤30%. BL, baseline; W, watts. Image modified from [52].

Figure 9 provides the plotted measurements of compensatory reserve influenced by 45 min of running exercise with dehydration and the impact of subsequent rehydration [52]. With each bar representing a 3-min average measurement, a reduction in compensatory reserve was reported from a resting standing position ≥92% to 28% after exercise was terminated. It should be noted that the longer exercise duration (45 min vs. 20 min) vs the previous experiments should have resulted in greater dehydration. Compensatory reserve was restored to approximately 60% within 10 min of the cessation of metabolic load created by the exercise and continued to recover to baseline levels of >90% as fluid ingestion reversed the absolute hypovolemia created by prolonged exposure to physical exercise with an unknown amount of dehydration. In this regard, a measurement of compensatory reserve provided an accurate integrated indicator of the individual’s physiological status and a way to assess recovery from heat stress and dehydration.

### 3.3. Future Capabilities Required to Further Advance the CRM

Although the CRM has been validated in its ability to track physiological changes in many different scenarios, it is potentially limited for use in humans in ambulatory or field settings in its current form, as it requires the use of either a noninvasive continuous blood pressure monitor (i.e., Finapres) or a transmissive (T) PPG sensor [14]. Continuous noninvasive blood pressure systems, while used in clinics and research labs, are far too bulky and expensive for an individualized monitoring device. Additionally, the TPPG sensor type is generally considered too obtrusive for wear-and-forget use, as it has to clamp over the recording site, which is most often a finger or sometimes an earlobe [54]. These locations are likely motion sensitive or hindering to the wearer. Moreover, many commercially available TPPG (or pulse oximeter) devices such as those frequently seen in hospitals and clinics have substantial filtering and automatic gain control built in, forcing the waveforms to be smoothed and homogenized. While this is optimal for their designed function of providing heart rate and SpO2 measurements, rich waveform information that could be used to estimate CRM is lost. The CRM has also not yet been validated in the presence of motion artifacts and external vibrations that will likely degrade the recorded arterial waveforms. A ruggedized wear-and-forget form factor is much more likely to be widely adopted for longitudinal monitoring for occupational, military and sports use [13,14]. Thus, a later section of this review will examine emerging wearable mechanical sensors, such as the seismocardiogram (SCG), which should be able to provide complementary or additional information to expand upon the current CRM. The SCG records the acceleration of the chest wall due to heart contraction and blood ejection movements as valves open and close.

One advantage of adding SCG signals to the CRM could be to decouple changes in the signals used for deriving CRM that are related to peripheral effects—e.g., vasodilation and altered vascular stiffness—from changes that are related to central effects—e.g., reduced preload. The substantial reduction in CRM for the hyperthermic individuals in Figure 6 (left) vs Figure 6 (right) even at 0 mmHg of LBNP suggests that peripheral vasodilation (from the hot environment) may be confounded to some extent with reduced compensatory reserve. PPG signals are very sensitive to ambient temperature and skin temperature in their waveform characteristics [55,56], and thus the combination of PPG (a peripheral measure) with SCG (a central measure) might be advantageous in future work to predict cardiovascular instability in individuals exercising in the heat. Finally, though the CRM has presented a convenient single metric to encapsulate a patient’s cardiovascular status, it utilizes a black-box deep learning approach for waveform comparisons that does not provide a direct linkage between algorithm features and physiological phenomena. An example feature that could be extracted for use with the current setup is measurement of arterial oxygen saturation, which will vary due to changes in altitude or sickness and could be combined with compensatory reserve [57].

### 3.4. Blood Volume Decompensation Status: Multi-Sensor Fusion with Explainable AI

**Figure 10:**
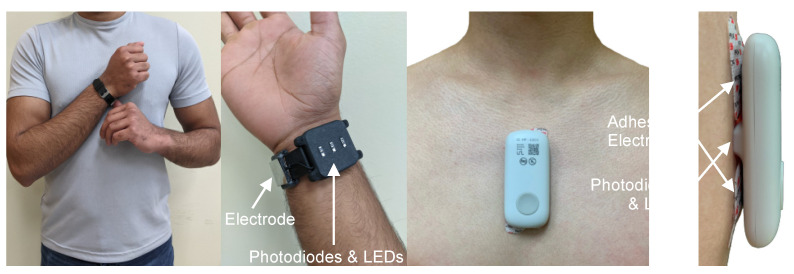
Device form factor. Electrodes for a single-lead ECG, photodiodes and LEDs to record the PPG, and tri-axial accelerometers and gyroscopes (internal to the devices) to acquire the SCG signal can be customized and modularized to work in multiple form factors. The left side shows the watch-based approach described in [60], while the right side shows an updated version of the chest-worn patch originally described in [59].

Encouraged by the results from CRM, a collaboration led by Inan and colleagues recently developed the blood volume decompensation status (BVDS) metric [12,58]. The goal of the BVDS metric builds from that of the CRM—to develop a single metric that represents the integrative compensatory response based on some aspect of PPG feature changes, and thus can be used to represent an individual’s compensatory reserve or decompensation status. One main difference between the BVDS and the CRM algorithmic approach is that the BVDS approach makes use of multi-modal cardiovascular sensing. A second main difference is that the BVDS approach leverages explainable AI approaches such that the exact features of the waveforms driving the output result can be individually examined from a physiological perspective. Thirdly, rather than using a TPPG sensor, the BVDS was developed with a reflectance-mode photoplethysmogram (RPPG) sensor, which can be placed anywhere on the body. Beyond capturing vascular information, electromechanical information from the heart is integrated into the BVDS metric by recording the electrocardiogram (ECG) and seismocardiogram (SCG) signals. This customized and modular sensing system design including ECG, SCG and RPPG sensors can be deployed in a wearable patch or smartwatch as shown in Figure 10 [59,60].

**Figure 11:**
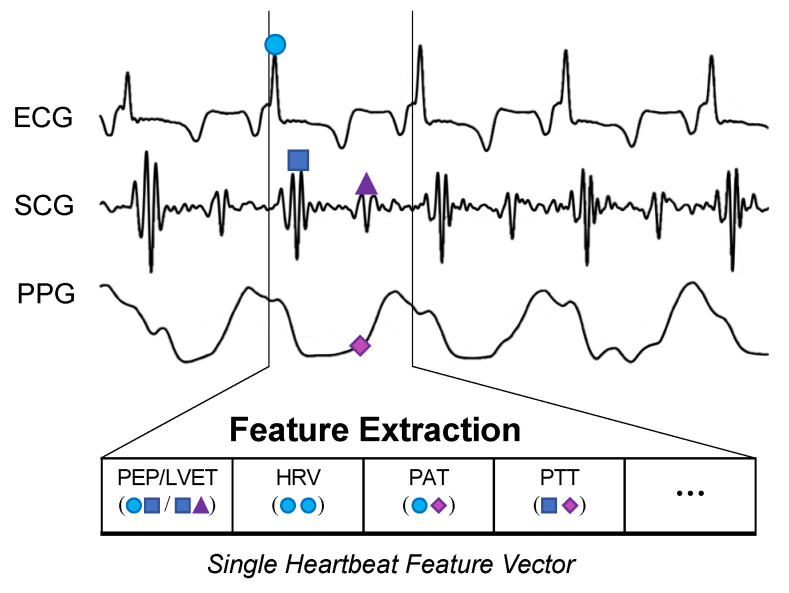
BVDS feature extraction. While the CRM evaluates 30-s segments of the recorded arterial waveform signal, the BVDS metric uses the ECG to segment and analyze all signals on a heartbeat-by-heartbeat level. Fiducial points are detected in each heartbeat and used to calculate cardiac timing intervals and a handful of other clinically relevant features.

**Figure 12:**
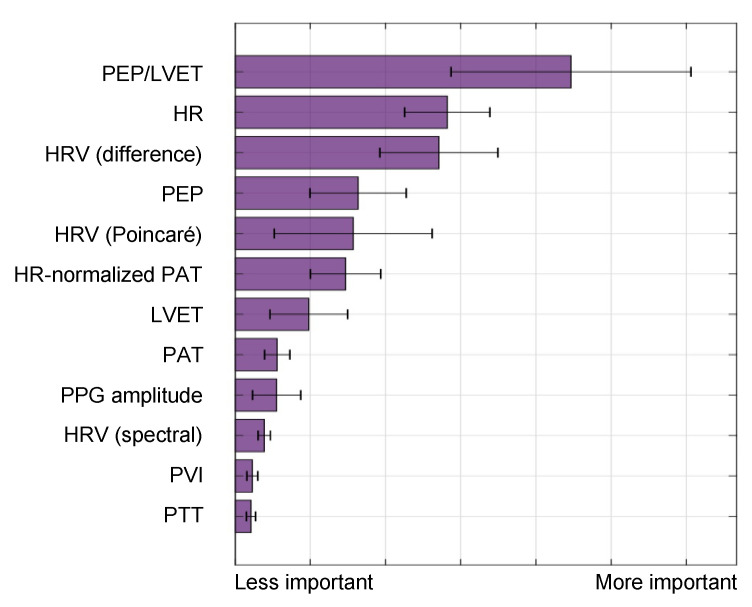
Feature importance for the BVDS model, as output by the random forest algorithm in [12]. Electromechanical features include the pre-ejection period (PEP), left ventricular ejection time (LVET) and their ratio, PEP/LVET along with heart rate (HR) and multiple measures of heart rate variability (HRV). Vascular features include the distal (and normalized) pulse arrival time (PAT), the distal pulse transit time (PTT), the PPG amplitude and the plethysmograph variability index (PVI). PEP/LVET is the most important feature for this model by a large margin, and six of the top seven features are from an electromechanical signal. This result highlights the relevance of including the ECG and SCG signals in predicting cardiovascular decompensation. Image modified from [12].

The BVDS metric has thus far been limited to a single preclinical animal (pig) study and is thus at an earlier stage of development. In this study, the animals underwent both relative and absolute hypovolemia through graded vasodilation and hemorrhage, as well as resuscitation with whole blood. ECG, SCG and RPPG were recorded continuously through the experiment. As shown in Figure 11, the inclusion of the ECG allows for feature extraction on a heartbeat-by-heartbeat level. A limited set of clinically relevant features was extracted from the ECG, SCG and RPPG signals. This set includes the pre-ejection period (PEP) and left ventricular ejection time (LVET) cardiac timing intervals, their ratio (PEP/LVET), the RPPG pulse arrival time (PAT) and pulse transit time (PTT), the plethysmography variability index (PVI) and RPPG amplitude, as well as heart rate (HR) and heart rate variability (HRV) measures. An initial model was developed using only the hemorrhage data recorded from the noninvasive sensors and compared to another model created with an analogous feature set extracted from simultaneously acquired invasive catheter blood pressure waveforms [58]. The BVDS model was further developed as data from the relative and absolute portions of the experiment were used together to train the random forest regression model with leave-one-subject-out cross validation to create a more generic metric of decompensation status [12]. The feature importance output by this model is shown in Figure 12, indicating that electromechanical features of cardiac performance were the most important predictors. This result shows that the ECG and SCG signals contain information that is very relevant to decompensation status or compensatory reserve. In particular, the ratio of PEP/LVET was the most important feature. In the literature, PEP/LVET has been shown to be a clear indicator of left ventricular performance [61,62] and changes in PEP/LVET have been shown to correlate with different stages of lower-body negative pressure [63].

### 3.5. Validation of Decompensation Status

**Figure 13:**
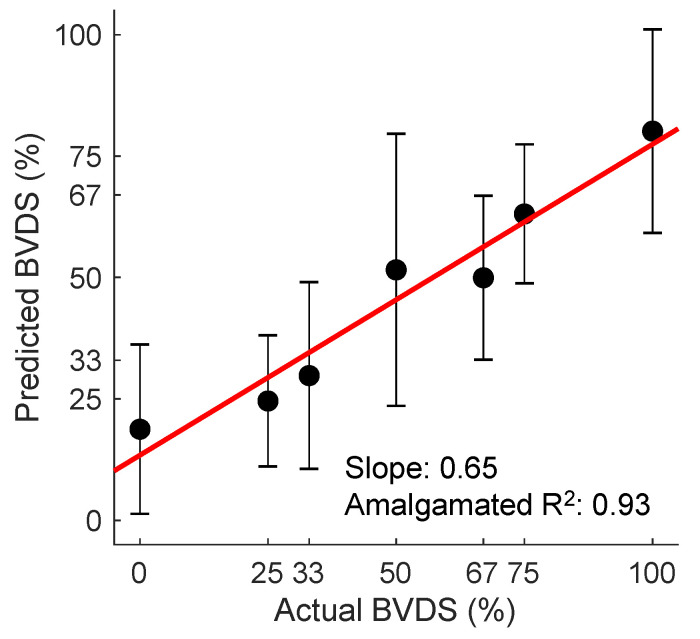
The BVDS metric performance in predicting decompensation. The line of best fit through the mean of the aggregated predictions for all animals during all portions of the experiment is shown in red. BVDS levels range on a scale from 0 to 100, with 100 indicating full decompensation status. The slope of the line (0.65) is an indicator of the overall prediction accuracy, while the R2 value of 0.93 is an indicator of the prediction consistency between BVDS levels. Standard deviation bars are also shown for each level, indicating the consistency of predictions within a single decompensation level. Image modified from [12].

Although the BVDS metric has only been validated in a single study thus far, it has shown promise as a globalized metric for predicting decompensation status in both relative and absolute hypovolemia as well as for resuscitation with whole blood following hemorrhage. The overall prediction results from this study are shown in Figure 13. In this figure, all predictions for all heartbeats for all pigs over the course of the entire protocol (baseline, relative and absolute hypovolemia and resuscitation) are aggregated. The mean and standard deviation for all graded decompensation status levels are shown, as well as the line of best fit through the means for each level. Status levels were defined such that 0% represents a baseline period and 100% represents full cardiovascular decompensation. Intermediate gradations were designated based on the percentage of blood removed during the hemorrhage portion of the experiment. As this model was created with data from separate interventions for relative and absolute hypovolemia and used to predict on hypovolemic, resuscitation and baseline periods, this represents a more generalized metric of cardiovascular decompensation status as compared to the previously published result focused on absolute hypovolemia alone.

**Figure 14:**
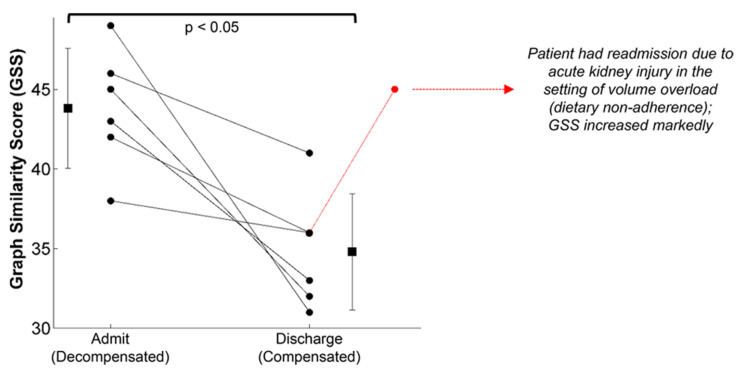
The graph similarity score representing structural differences in the SCG signal recorded from a wearable patch found significant differences between compensated and decompensated heart failure patients from admission to discharge. Though all patients improved following treatment, some patients responded much better to the treatment than others. Image from [64].

The wearable chest-worn patch [59] that provides measures of simultaneous ECG, SCG and PPG (and thus could be used to monitor BVDS) has been used in other studies that are relevant to our discussion here. The most pertinent data may have been generated in a study designed to segregate patients with compensated and decompensated heart failure [64]. In heart failure, patients are generally hypervolemic rather than hypovolemic, while still experiencing poor circulation and perfusion. Additionally, environmental stressors and exercise exacerbate their poor cardiac performance, particularly for patients with decompensated heart failure. In this study, the structure of the SCG signals recorded with the patch was studied in 45 patients with heart failure before and after a standard six-minute walk test, after which a similarity score was computed from the graph representing the structure of the SCG data in the spectral domain. As seen in Figure 14, significant differences in the SCG signal structure were found between decompensated heart failure patients at admission and at discharge after receiving treatment. It is notable that some patients responded much better to the treatment than others, again highlighting the need for individual-specific metrics of performance. Specifically, decompensated patients had a higher graph similarity score comparing their SCG before and after the walk test than did compensated patients, indicating a higher similarity in contractility and cardiovascular hemodynamics between rest and recovery, meaning their cardiovascular systems were unable to compensate for the strain of exercise. In short, the decompensated patients experienced a lower compensatory reserve than compensated heart failure patients. In turn, we would expect heart failure patients (and those who are yet to be diagnosed) to experience a lower operating compensatory reserve and a faster decline of their reserve than healthier patients for a similar amount of physical activity, including activity in the workplace.

### 3.6. Advancements and Next Steps

The BVDS metric requires validation in additional studies. As the initial algorithm was developed in an animal model, new datasets should be curated from human subjects with realistic progression of perturbations that includes both relative and absolute hypovolemia. Realistic noise sources should also be included in this development in the form of data from both free-moving subjects and those being transported in multiple classes of vehicles. Advanced modeling techniques, such as the graph analysis described for the heart failure study, and other techniques ,such as transfer learning and time series analysis, should be explored.

The previously constructed wearable sensing patch for ECG, SCG and environmental context sensing was designed for use in patients with heart failure—a frail population of older patients that would wear the device around the home and during normal activities of daily living. To enable usage of this patch for wearable sensing in the context of human performance—i.e., occupational, military, and sports applications—the hardware should be ruggedized, appropriately miniaturized, and validated with a broad range of environmental testing scenarios. For example, the hardware and adhesives must be designed to tolerate high moisture levels such as heavy sweating. The mechanical coupling integrity of the sensing system to the chest should be evaluated at high levels of vibration that could result from motion artifacts or other external vibration sources, as well as in the presence of fluids such as sweat or blood. To this end, some initial testing and validation work has been conducted at Georgia Tech with healthy human subjects performing various exercise tasks both indoors and outdoors, and with signals being measured in the presence of external vibrations [65,66,67].

When it comes to addressing motion artifacts, there are two main stages that should be considered. The first is developing customizable signal quality indices (SQI) to remove portions of the recording that contain too much noise. The second stage then takes the output from the SQI and processes the signal in the presence of remaining noise. ‘Motion artifacts’ include any noise sources related to the user—physical movement, speech, interference from clothing or gear, etc. The SCG and PPG have been criticized for their susceptibility to motion artifacts; however, recent studies demonstrate that PPG and SCG can be ruggedized for free-range use. Clifford and colleagues have developed quality indices for hospital-grade ECG and PPG signals and shared them through their open-source cardiovascular waveform toolbox on PhysioNet [68]. An SQI developed specifically for SCG signals (but that can be applied to other signals such as PPG) is presented in [69]. This study retroactively stratified SCG heartbeat quality recorded from subjects during rest, exercise and recovery. Heartbeats from the SCG were segregated based on their similarity to a template beat, allowing for higher quality beats to be identified during the noisier periods such as exercise. Multiple groups have made progress on the second stage processing for SCG and PPG signals. Yang et al. utilized an adaptive filtering technique to effectively process SCG recordings in walking subjects [70]. Additional studies have indicated that using a gyroscope along with an accelerometer to record the SCG can improve signal feature estimations, possibly due to differing levels of noise in the linear and angular domains [71,72]. By including an array of sensors and leveraging independent component analysis, Yang et al. were able to extract relevant cardiac timing intervals from the SCG in both walking and jogging subjects, tested up to 4.6 mph [73]. Beyond SCG improvements, multi-wavelength PPG analysis shows promise for developing more robust feature extraction methods [60,74]. Adaptive filtering and signal deconstruction/reconstruction approaches have also been utilized for analyzing PPG recordings from subjects during moderate and intensive exercise [75,76].

The problem of reducing the impact of external vibration sources, such as vehicles, on SCG and PPG recordings has been less-thoroughly explored than the problem of reducing motion artifacts in general. One group recorded the SCG of a single subject for an entire day, including commuting to and from the office in a subway train [77]. To process the portions with subway noise, Di Rienzo et al. utilized an ensemble averaging approach prior to annotating the heartbeats. In a separate study, Lin et al. combined SCG recordings with accelerometer recordings taken on a subway train and used an ensemble empirical mode decomposition approach to remove the vehicular noise [66]. Similar approaches could potentially be used to remove noise from additional transport vehicles or other external vibration sources. A summary of the current state of the noise reduction stages (signal quality indexing, motion artifact and external vibration removal) is contained in Table 1.

## 4. Future Technology Advancement Opportunities

### 4.1. Contextual Measures

In terms of predicting specific physiological outcomes, additional context can help limit the number of false positive and false negative indicators output by the system. For example, activity recognition could keep the system from alerting to a supposed acute injury when the subject has simply climbed a long flight of stairs. The system could also use environmental context such as ambient temperature to better forecast a user’s reserve levels when the current level of activity is sustained in that temperature.

With the inclusion of an accelerometer into the BVDS design comes the potential to perform activity recognition and monitoring to provide context for compensatory reserve predictions. Exploration of accelerometer and IMU-based activity recognition has spanned systems with a single sensor [84,85,86] as well as systems with many sensors [87,88] in an effort to classify activities, such as rest, walking, running and cycling as well as to detect more discrete events, such as falling [89]. Other groups have estimated energy expenditure [90], with some groups utilizing additional sensors, such as barometers [91] or a pressure sensor in the shoe to evaluate the amount of weight born by the user [92]. Physiological features, such as heart rate, have also been added into the calculation for improved estimation [93]. However, Murakami et al. evaluated 12 different popular commercially available accelerometer-based devices in 2019 and concluded that more work is needed in the area of physical activity energy expenditure prediction for wearable devices [94].

An et al. recently published a method called AdaptNet, in which they leverage the triaxial accelerometer data from a chest patch for robust activity recognition. Using data from multiple domains, they accurately identified subjects standing at rest, walking on level ground, walking at a decline, and walking at an incline both with and without stairs [83]. Using this type of approach, a monitoring device could record the duration and perhaps metabolic intensity of the user’s physical activity [95] and evaluate changes in the compensatory reserve due to that activity level and perhaps other environmental stressors. A user’s individualized compensatory reserve change in response to a particular level of energy expenditure may then be learned by the system, and anomalous behavior deviating from this individualized response can potentially indicate performance degradation resulting from any number of factors, such as inadequate sleep, hydration, or nutrition. Periods of anomalous responses can then be alerted to the user prior to negative sequelae, resulting in setbacks in training regimens for athletes, for example, or higher risk of musculoskeletal injury from excessive fatigue.

We discussed how the impact of physical activity on the compensatory reserve can be adjusted due to environmental variables. Beyond activity recognition, additional context surrounding the environment as well as some more information from the user could be beneficial in estimating their true compensatory reserve. The first of these metrics is temperature [96,97]. Ideally, the user’s skin temperatures would be monitored from one or several sites, as well as ambient temperature [20]. Although temperature sensors are now sufficiently miniaturized and commonplace, accurately measuring these temperatures in practice using a wearable device or system is non-trivial due to mixed heating effects from multiple sources. Secondly, an altimeter or barometer could allow the device to keep track of physiological changes potentially due to altitude (hypoxia) or atmospheric pressure as well as improved activity recognition and energy expenditure.

### 4.2. Integration

We envision a future that incorporates elements of the CRM with advances in sensing technology, multiple modalities of cardiovascular sensing (as seen in the BVDS metric), additional contextual measures and advanced noise reduction algorithms into a fully wearable system, creating a robust and physiologically sound estimation of the free-living user’s compensatory reserve or decompensation status during physical work and environmental exposure. Such a system would enable quantification and management of previously discussed hypovolemia issues to optimize health and performance. This wearable system could consist of a chest-worn patch, though it could also be contained in a watch, or include additional sensors and hardware in occupation-specific gear. If the entire system is contained in a watch, it should be noted that all signal measurements would not be fully continuous—the user would take measurements intermittently throughout the day during rest periods while pressing the watch against their sternum (as seen in Figure 10) to record the ECG and SCG. Though leading to fewer readings throughout the day, one benefit of this approach is an appreciable reduction in motion artifacts. Alternatively, a benefit of the complete wear-and-forget form factor such as the chest patch allows for continuous measurements, including while the subject may be unconscious due to regular sleep or injury. To be fully useful, this system would require a method for giving feedback to the user about their physiological state in an effort to prevent dehydration and heat stress, or to a supervisor or health practitioner for other use cases, such as injury. Recent developments in wearable sensing can allow for improved and increasingly viable form factors for the next generation of devices used to predict a user’s compensatory reserve. For example, advances in biosignal-specific integrated circuits [98] as well as flexible and stretchable circuitry [99] motivate the design of smaller, less-obtrusive and more comfortable wearable devices.

**Figure 15:**
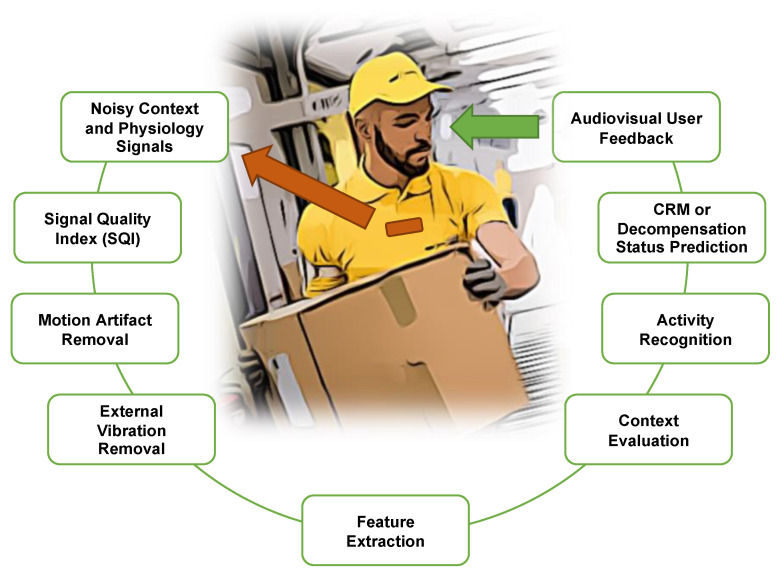
Processing stages in an example use case. Noisy signal recordings from a ruggedized chest-worn sensor (brown) go through a signal quality assessment and then motion and external vibration removal prior to feature extraction. Once high-quality features are extracted from the signals, predictions and evaluations of context, activity and reserve or decompensation status can be made. This summarized information is then relayed back to the user.

Figure 15 shows a futuristic scenario with a worker wearing a device on the chest that is capable of measuring the advanced CRM or BVDS metric. The processing steps described in prior sections that are required for continuous measurements from a free moving subject are shown in the green boxes. First, signal recordings from the device go through a series of noise reduction steps that include a determination of signal quality as well as external vibration and user-generated motion artifact removal. The required features for the predictive model can then be extracted from the clean signals. These features are then fed to the prediction pipeline that includes evaluation of context and activity recognition before estimating the user’s compensatory status and providing feedback on that status to the user.

To create this vision of the next level of compensatory reserve or decompensation status models, all required sensors first need to be combined into a single wearable or realistic system of wearable devices. Challenges, such as capturing the temperature correctly in the face of multiple heat sources (including the device itself), need to be addressed. Additionally, there are challenges with processing and storing data from multiple sensors and questions about how to power the device(s), along with decisions to be made about how to interact with the user and others appropriately to maintain patient privacy.

It is important to mention that a multitude of machine learning models have been developed to predict undesirable patient states with regards to hemorrhage detection and general cardiovascular instability. For example, support vector machines have been used in noninvasive estimates of simulated and actual hemorrhage severity [100,101], though it should be noted that these models were used to separate 2–3 classes of severity rather than have a continuous output and were also not individual-specific. Other studies have utilized random forest, support vector machines, k-nearest neighbors and neural network algorithms to model risk of cardiorespiratory instability in the hospital through integrated monitoring systems [102,103,104]. K-nearest neighbors, random forest, gradient boosted trees and logistic regression with L2 regularization were used to classify hypotensive events in time series data from the ICU [105]. However, all of these models rely heavily on standard vital signs (e.g., blood pressure, heart rate, SpO2, respiratory rate) acquired in the hospital setting, some of which come through invasive measures. Though vital signs have proven to be inferior in sensitivity and specificity compared to measures of the compensatory reserve regarding their application to wearable systems [14], the CRM and BVDS metrics currently report patient status in the moment and do no look-ahead forecasting of compensatory states, which could be extremely beneficial for the user and healthcare personnel.

In parallel with developing the physical system, advanced machine learning models leveraging approaches such as graph analysis, transfer learning and time series analysis will need to be developed that incorporate both the CRM and BVDS methods along with additional contextual measures. To be most effective, these models will need to be created from rich datasets that are curated from a diverse population performing multiple tasks in austere environments. The datasets must contain real physiological challenges, true to life sensor noise, and gold standard reference measures for the physiological challenges.

### 4.3. Applications and Opportunities

Heat strain is a common problem for workers, warfighters and athletes due to a combination of performing vigorous physical work, exposure to environment heat, and/or wearing heavy clothing, uniforms or protective equipment [20]. Currently, occupational and military communities measure heat strain in workers and warfighters by monitoring or estimating core temperature alone or with heart rate [9,10]. This is because it was previously believed that if core temperatures were maintained <38.5 °C, most workers can complete a work shift and that ~39.2 °C was the upper limit of safe physiological tolerance [106,107,108,109]. Unfortunately, such general guidance does not hold for individuals. Many individuals performing physical work can well-tolerate higher core temperatures (>40 °C) without impairing work capacity or health, especially when the skin is cool [20,110]. Likewise, if the skin temperatures are fairly high, core temperature tolerance can be 38.5 °C or lower in some individuals [111]. For example, individuals working at moderate metabolic rates (that are common for occupational and military tasks) with high skin temperatures (such as when wearing protective clothing or in hot-humid conditions) there is a normal bell distribution between core temperature and incidence of heat exhaustion [111]. Likewise, combining heart rate with core temperature measures does not provide a reliable safety index of an individual’s tolerance to exercise-heat strain [10]. When monitoring free-living workers, heart rate can be influenced by work intensity, isometric tasks and other confounding factors beyond heat strain or dehydration. As discussed, a primary physiological mechanism impairing physical work capacity with heat strain and dehydration is often cardiovascular in origin [20,24]; thus, it is logical to monitor each individual’s compensatory reserve or decompensation status rather than set core temperature or heart rate thresholds to manage heat strain exposure.

As discussed, hypovolemia from dehydration or environmental exposure and/or physical work can adversely affect health and performance, thus motivating the need for continuous physiological monitoring. We argue that the estimation of cardiovascular strain should incorporate mechanical measures that can include PPG and SCG signals such that the effects of reduced preload on cardiac performance can be more directly assessed. Moreover, single vital sign or feature-based approaches are insufficient, and thus machine-learning techniques should fuse multiple waveform features to capture the complex nature of the physiological response to hypovolemia. For example, blood pressure itself may be often regulated until physical or heat exhaustion, while arterial and pulse pressure instabilities or altered cardiomechanics might result much earlier due to afferent signaling representing baroreceptor unloading and changes in tissue perfusion and cardiac filling and vascular resistance. By sensing these cardiovascular instabilities, one could then predict impending physical exhaustion beforehand, which is what Convertino and colleagues [7] demonstrated to occur with hemorrhage prior to cardiovascular collapse (loss of blood pressure regulation) or accompanying perturbations of dehydration and heat stress with orthostatic challenges [51].

Likewise, diarrhea and vomiting induced dehydration are serious military and civilian problems during both combat deployments and humanitarian missions as they are a major cause of mortality in developing nations. Though a robust wearable system for measurement of compensatory reserve or decompensation status would not directly measure hydration status [16], monitoring the associated circulatory impact of this perturbation will provide a proxy measure [51]. As individuals consume or are administered fluids, the vascular volume will be restored and improve cardiovascular stability, resulting in recovery from dehydration—or if combined with body cooling recovery from hyperthermia, and a measure of compensation or decompensation could indicate how close the person is to “full recovery” from either these individual or combined hypovolemia stressors. Thus, a compensatory reserve or decompensation measure would provide objective individualized guidance regarding work–rest ratios and recovery break management.

Physiological adaptation to physical work (physical training) and heat stress (heat acclimation) both require blood volume expansion and central hemodynamic changes to overcome the vasodilation challenges and maintain cardiovascular stability associated with improved work capabilities [25,38,39]. Stressor adjustment decisions for physical training and heat acclimation (whether to increase training intensity or heat stress exposure) are often based on work performance improvements and/or easily measured vital sign reductions for a given stress. Determining work performance improvements usually requires separate standardized evaluations (with identical conditions) and there is often debate over which easily measured vital sign provides the most effective index [112]. A measure of cardiovascular stability and decompensation, during any non-standardized condition, would provide an important integrated measure regarding the compensatory status needed to support the task and monitor training and acclimation adaptations.

## 5. Summary

The manuscript provides several examples of occupational, military and sports medicine hypovolemia problems that could be managed with wearable technology and machine learning for optimizing health, safety and physical work capabilities. We have discussed the biological rationale for compensatory reserve and decompensation status and shown their sensitivity to numerous hypovolemia perturbations in human and animal models. We have highlighted recent technology advances that will enable this approach for wearable monitoring—decision aid systems for free-living workers in austere conditions. Finally, we have described the needed technology and algorithm advances to make effective individualized management of hypovolemia for a variety of common occupational, military and sports applications.

## Figures and Tables

**Table 1:** Summary of Ruggedization Metrics.

Ruggedization Measure	Purpose	Major Challenges	State of the Art	Opportunities for Advancement
Signal Quality Indexing	Assess noisy portions of the signal to determine whether they are salvageable for feature extraction	Determining an appropriate comparator (i.e., template) to recognize “good” signals, particularly in changing physiology	[68,69]	Address specific wearable issues: sensor misplacement; realistic varied noise sources such as clothing interference, speech, body movement, fluids, etc.
Motion Artifact Removal	Mitigate the effects of noise due to movement of the subject	Tracking physiology during periods of extended motion	[78] (ECG) [76] (PPG) [73] (SCG)	Multimodal, multi-sensor analysis; realistic varied noise sources; sparse estimation;
External Vibration Removal	Mitigate the effects of noise from the environment such as from transport vehicles	Semi-periodic in-band noise sources	[79] (ECG) [66] (SCG)	Methods specific to reflectance-mode PPG; noise data from multiple vehicle types; multimodal, multi-sensor analysis
Moisture Resistance	Protect sensors and signals from fluid interference (e.g., sweat, blood, mud)	Fluids can block or short sensors and distort signals	[80,81]	Improved packaging; machine learning or signal processing methods for detecting and adjusting to fluid interference
Activity Recognition	Provide context for physiological measurements	Large amounts of data required with consistent labeling	[82,83]	Multi-modal and additional domain transfer techniques

## Data Availability

Not applicable.
